# Supplementary material for: Impact of self-efficacy and burnout on professional development of physical education teachers in the digital age: a systematic review
Source: PeerJ. 2025 Feb 21;13:e18952. doi: 10.7717/peerj.18952 (PMC11849512; doi:10.7717/peerj.18952)
Supplement: Supplemental Information 3 [file peerj-13-18952-s003.docx]

**Systematic Review and/or Meta-Analysis Rationale**

**For systematic reviews/meta-analyses, authors need to provide the following information:**

**1. The rationale for conducting the systematic review/meta-analysis;**

**There is a notable shift from the traditional face-to-face mode of teaching to a blended approach in regular physical education. In this transition, digital technology plays a pivotal role. Hence, physical education (PE) teachers must master digital technology skills. Concurrently, the advancement of digital technology necessitates the active participation of PE teachers in ongoing professional development. An investigation into the factors affecting professional development identified self-efficacy and burnout as having a significant impact.**

**The study revealed that burnout exerts adverse effects on teachers, including physical and mental health concerns, a lack of focus, and limited opportunities for self-reflection(Fathi J 2021; Hershko-Leibovits 2021), thereby hindering professional development(Hershko-Leibovits 2021; Pereira et al. 2022). Additionally, PE teachers with low self-efficacy displayed diminished effectiveness in their professional development and lower motivation(Rombaoa Tanaka et al. 2020). Surprisingly, no literature was discovered during the literature review or at the time of registration that had synthesized these aspects, prompting the necessity of a systematic review focused on understanding the impact of digital self-efficacy and burnout on the professional development of PE teachers.(Xiong et al. 2019)**

**It is of utmost importance to comprehensively address the obstacles to digitalization when promoting digital transformation in education. Furthermore, as the front-line implementers of digital transformation, PE teachers play a pivotal role. A systematic analysis of their professional development competencies and strategies to mitigate burnout and enhance self-efficacy can significantly aid educators in honing their craft. As a result, this systematic review delves into digital self-efficacy, burnout, and factors impeding the professional development of physical education teachers, providing valuable insights for educators and researchers alike.**

**2. The contribution that it makes to knowledge in light of previously published related reports, including other meta-analyses and systematic reviews.**

**1. Burnout did increase during the COVID-19 pandemic. However, compared to individuals employed in other career fields, K - 12 teachers experienced little difference in levels of stress and burnout. Future research needs to include more longitudinal designs and examine the interactions between individual and organisational characteristics in developing teacher stress and burnout during the COVID-19 pandemic and beyond (Westphal et al. 2022).**

**2. Among teachers, physical education teachers are particularly understudied, although a recently published systematic review found that they face high levels of stress. To better explore burnout syndromes among physical education teachers, this study conducted a systematic review that searched up to six languages (von Haaren-Mack et al. 2020).**

**3. The objective is to pinpoint the origins of stress, chronic stress-related outcomes, and factors influencing stress levels in physical education teachers. Additionally, we aim to provide recommendations for future research and teacher education in this context. In terms of examining the connection between stress sources and the enduring effects of chronic stress experienced by physical education instructors, forthcoming studies should (a) encompass the entire stress continuum, (b) employ more comprehensive stress measurement techniques, and (c) delve into potential stress-mitigating factors. From a practical standpoint, physical education teachers must receive thorough pre-service and in-service training to grasp the primary stressors associated with their profession and cultivate effective coping strategies** (Alsalhe et al. 2021).

**4. This paper centres its attention on elucidating the structural connection between burnout in the field of education and teacher self-efficacy. The primary aim is to gain a deeper comprehension of the elements that fuel burnout among K-12 educators, with the ultimate goal of offering actionable recommendations for mitigating and preventing burnout. Enhancing teacher self-efficacy can be a viable strategy to alleviate burnout. Substantial evidence underscores that a deliberate increase in teacher self-efficacy can not only reduce burnout but also foster unprecedented levels of student motivation and engagement in the future** (Stotz 2021).

**5. The objective of this study was to conduct a comprehensive analysis of literature concerning physical education teachers' self-perceived confidence in instructing general physical education, employing self-efficacy theory as a framework.** The main findings of the study indicate that perceptions of training, amount of experience, and staff support significantly influence teachers' self-efficacy in teaching students with disabilities (Nowland & Haegele 2023).

**6. This study was conducted to investigate the influence of self-efficacy among primary and secondary school teachers on their utilization of assistive technology. Furthermore, this review seeks to provide a concise overview of the historical context of self-efficacy within the realm of inclusive education in general while also delving into the assessment instruments, sample selection, and the validity and reliability measures employed in the selected studies. In the future, trainers prioritise professional development initiatives designed to bolster alterations in teachers' self-efficacy, ultimately leading to more effective incorporation of assistive technology in their educational practices** (Giek 2021).

7. Teachers are susceptible to burnout, and numerous studies have highlighted teacher burnout as a key contributor to teacher turnover. It has been proposed that self-efficacy serves as a protective shield against burnout. A noteworthy correlation was observed between classroom management self-efficacy (CMSE) and the three dimensions of burnout, signifying that teachers possessing higher levels of CMSE were less prone to burnout. The paper also delves into practical implications and provides recommendations for prospective research in this area (Aloe et al. 2014).

8. Insufficient capacity for digital professional development of teachers, including digital literacy and ICT skills, by 2018 (Fernández-Batanero et al. 2022).

9. Virtual reality (VR) experiments have become prevalent in physical education and training, offering substantial support in enhancing decision-making abilities, such as prediction skills. The primary objective of this study is to provide a comprehensive understanding of the current landscape of virtual reality applications in sports. This investigation aims to determine the most effective methods for integrating virtual reality into physical education and training. The utilization of VR in these areas demonstrates a noticeable upward trajectory. Notably, the predominant approach for implementing VR in physical education and training involves the use of head-mounted displays (HMDs) in conjunction with motion capture systems (Putranto et al. 2023).

**10. The primary discovery of this study revolves implementation quo in the blended teaching model in the context of physical education. Furthermore, the research revealed five distinct challenges and emerging trends related to the utilization of the blended teaching model in the field of physical education. These challenges encompass instructional design, technical proficiency, self-regulation, feelings of detachment and isolation, and changes in beliefs** (Wang et al. 2023).

Based on previous reports, the research highlights of this review are as follows:

1. burnout and self-efficacy integration studies have focused on K-12 teachers(Aloe et al. 2014; Stotz 2021; Westphal et al. 2022) and have not singled out physical education teachers. Instead, this review explored burnout and self-efficacy among physical education teachers based on prior reports.

2. Related reports of professional development have been limited to self-efficacy (Nowland & Haegele 2023) or burnout (Alsalhe et al. 2021; von Haaren-Mack et al. 2020); however, no research has explored the integration of these two dimensions in the direction of sports.

3. based on digital technologies, previous reports of the main sports-orientated virtual reality technologies (Giek 2021; Putranto et al. 2023), blended teaching models(Fernández-Batanero et al. 2022; Putranto et al. 2023; Wang et al. 2023), while this review focuses on understanding the effects of burnout and self-efficacy in digital professional development during the exploration of digital technologies.

**In conclusion, this systematic review carries significant research implications as it seeks to revitalize our understanding of the factors influencing the digital professional growth of physical education teachers, particularly in the realms of burnout and self-efficacy.**Despite the numerous previous studies on this topic, the ever-changing social context continuously influences the dynamics of burnout and self-efficacy. Therefore, an investigation into the influence of burnout and self-efficacy on professional development within the digital landscape can usher in fresh perspectives and steer the course of digital professional growth.

Aloe AM, Amo LC, and Shanahan ME. 2014. Classroom management self-efficacy and burnout: A multivariate meta-analysis. *Educational psychology review* 26:101-126.

Alsalhe TA, Chalghaf N, Guelmami N, Azaiez F, and Bragazzi NL. 2021. Occupational burnout prevalence and its determinants among physical education teachers: A systematic review and meta-analysis. *Frontiers in Human Neuroscience* 15:553230.

Fathi J GV, Derakhshan A. 2021. Self-efficacy, Reflection, and Burnout among Iranian EFL Teachers: The Mediating Role of Emotion Regulation. *Iranian Journal of Language Teaching Research*:9(2),13-37. 10.30466/ijltr.2021.121043

Fernández-Batanero JM, Montenegro-Rueda M, Fernández-Cerero J, and García-Martínez I. 2022. Digital competences for teacher professional development. Systematic review. *European Journal of Teacher Education* 45:513-531.

Giek CB. 2021. A Systematic Review of the Influence of Teacher Self-Efficacy on the Use of Assistive Technology.

Hershko-Leibovits N. 2021. Teacher Burnout: An Examination of the Relationships Between Professional Development, Self-Efficacy and EnthusiasmMaster's. University of Haifa (Israel).

Nowland LA, and Haegele JA. 2023. The Self-Efficacy of Physical Education Teachers to Teach Students With Disabilities: A Systematic Review of Literature. *Adapted physical activity quarterly* 1:1-23.

Pereira ECDCS, Ramos MFH, and Ramos EMLS. 2022. Síndrome de burnout e autoeficácia em professores de educação física. *Revista Brasileira de Educação* 27. 10.1590/s1413-24782022270045

Putranto JS, Heriyanto J, Achmad S, and Kurniawan A. 2023. Implementation of virtual reality technology for sports education and training: Systematic literature review. *Procedia Computer Science* 216:293-300.

Rombaoa Tanaka N, Boyce LK, Chinn CC, and Murphy KN. 2020. Improving Early Care and Education Professionals’ Teaching Self-Efficacy and Well-Being: A Mixed Methods Exploratory Study. *Early Education and Development* 31:1089-1111. 10.1080/10409289.2020.1794246

Stotz AK. 2021. Structural Relationships Among Teacher Self-Efficacy and Burnout.

von Haaren-Mack B, Schaefer A, Pels F, and Kleinert J. 2020. Stress in physical education teachers: a systematic review of sources, consequences, and moderators of stress. *Research Quarterly for Exercise and Sport* 91:279-297.

Wang C, Omar Dev RD, Soh KG, Mohd Nasirudddin NJ, Yuan Y, and Ji X. 2023. Blended learning in physical education: A systematic review. *Frontiers in public health* 11:1073423.

Westphal A, Kalinowski E, Hoferichter CJ, and Vock M. 2022. K− 12 teachers' stress and burnout during the COVID-19 pandemic: A systematic review. *Frontiers in Psychology* 13:920326.

Xiong Y, Sun XY, Liu XQ, Wang P, and Zheng B. 2019. The Influence of Self-Efficacy and Work Input on Physical Education Teachers' Creative Teaching. *Front Psychol* 10:2856. 10.3389/fpsyg.2019.02856
